# Impact of Body Mass Index on Gastrointestinal Disorders: A Cross-sectional Study in a Pakistani Population

**DOI:** 10.7759/cureus.7722

**Published:** 2020-04-18

**Authors:** Shahid Ahmed, Hafeezullah Shaikh, Sajjad Jamil, Hamid Ali, Maryam Abbasi

**Affiliations:** 1 Internal Medicine, Darul Sehat Hospital, Karachi, PAK; 2 Gastroenterology, Zubaida Medical Centre, Karachi, PAK; 3 Gastro, National Institute of Liver GI Diseases (NILGID), Dow University Hospital (DUH), Karachi, PAK; 4 Gastroenterology, Liaquat National Hospital & Medical College, Karachi, PAK; 5 Miscellaneous, Darul Sehat Hospital, Karachi, PAK

**Keywords:** body mass index, gerd, obesity, gastritis, duodenal ulcers

## Abstract

Background: The prevalence of obesity is on the rise globally. Pakistan ranks ninth out of 188 countries in terms of obesity. Literature regarding any potential role of obesity in gastrointestinal manifestations is limited. Besides, scarce information is available on possible connection between obesity and gastrointestinal pathology. This research, therefore, explores the impact of body mass index (BMI) on gastrointestinal symptoms and endoscopic discoveries.

Methods: A cross-sectional study was conducted at Darul Sehat Hospital, Zubaida Medical Centre, and Liaquat National Hospital & Medical College, Karachi, Pakistan from 1^st^ July 2017 to 30^th^ November 2018. Patients scheduled to undergo endoscopy were surveyed before the procedure in which they were asked about demographics, comorbid, and gastrointestinal manifestations. The association between BMI and endoscopic findings and related gastrointestinal symptoms was assessed using Pearson chi square test and binary logistic regression. The significance level was set at 0.05.

Results: A total of 2148 subjects were included in the study out of which 20.2% were overweight (BMI 23-24.9) and 20.9% subjects were obese (BMI >25). Both the gastrointestinal symptoms and endoscopic findings were found to be significantly associated with the BMI (p<0.05 for both). Moreover, binary logistic regression revealed obesity to be a significant contributor of abnormal endoscopic findings (adjusted odds ratio, AOR, 2.93; 95% confidence interval, CI, 2.35-3.65).

Conclusion: Based on the study results, obesity was identified as a risk factor associated with gastrointestinal symptoms like gastro esophageal reflux disease (GERD) and other related gastrointestinal conditions such as erosive gastritis, duodenal ulcers, reflux esophagitis, and large hiatal hernia.

## Introduction

The prevalence of obesity is on the rise globally. According to World Health Organization, per capita weight has dramatically increased worldwide since 1980 and in 2016, more than 1.9 billion people aged 18 years or above were overweight while more than 600 million grown-ups were obese worldwide [1]. In Pakistan, increased weight has become a significant danger to clinical wellbeing of individuals and as indicated by the Global Weight of Disease study, Pakistan is positioned ninth out of 188 nations in terms of obesity [2]. Obesity is considered a risk factor for many noncommunicable diseases such as diabetes, hypertension, coronary illness, stroke, and gall stones [3-5]. Moreover, as indicated by The American Institute for Cancer Research there is persuading proof for a connection between obesity and a few types of cancers like esophageal cancer, liver malignancy, postmenopausal breast tumor, gastrointestinal cancers, and colorectal malignancies [6].

Any potential role of obesity in the development of gastrointestinal symptoms is unclear and literature reveals equivocal findings in this regard. Increased body mass index (BMI) has earlier been reported to be associated with upper abdominal pain, gastro esophageal reflux, and indigestion but not with lower abdominal pain, nausea, and vomiting [7]. It is known that the occurrence of heartburn and regurgitation in obese individuals is due to excessive transient lower esophageal sphincter relaxation during the postprandial period, which results in elevated esophageal acid exposure [8]. Moreover data from a review published in 2013 demonstrate that weight reduction can result in resolution of gastro esophageal reflux ailment manifestations [9].

Other than upper gastrointestinal manifestations, any available data on a potential relationship between increased weight and other gastrointestinal pathology are scarce and conflicting [10-11].

The objective of the current study was, therefore, to assess the relationship between increasing BMI, gastrointestinal symptoms, and endoscopic findings in Pakistani population.

## Materials and methods

A prospective, cross-sectional study was conducted at Darul Sehat Hospital, Zubaida Medical Centre, and Liaquat National Hospital & Medical College, Karachi, Pakistan from 1st July 2017 to 30th November 2018. The calculated sample size using 3% prevalence, at 0.725% precision and 95% CI was 2123 and to accommodate for nonresponders this was inflated by 1%-2% and 2148 individuals, aged 18 years or above, finally consented to participate in this study. Individuals with any comorbidity like diabetes, hypertension and chronic liver disease or history of cancer, inflammatory bowel disease, celiac disease, history of Helicobacter pylori eradication were excluded from the study. Participants who had been medicated with acid suppressing drugs like H2 receptor blockers, proton pump inhibitors, and nonsteroidal anti-inflammatory drugs during the previous month were also excluded from our study.

Prior to endoscopy a questionnaire was administered in which patients were asked about demographics details such as age, height, and weight. Patients were also asked to score gastrointestinal symptoms over the past four weeks by a validated gastrointestinal symptoms rating scale (GSRS) [12]. In this questionnaire they were asked about specific symptoms like epigastric pain, heartburn, regurgitation, bloating, nausea, lower abdominal pain after a meal, after defecation, diarrhea, etc. They were further asked to rate the severity of these symptoms as no, mild, moderate, quite a lot, severe, very severe, and unbearable symptoms. The symptoms were then grouped into three categories: gastro esophageal reflux disease (GERD) (which was defined as heartburn and regurgitation), dyspepsia (which included symptoms of epigastric pain, abdominal bloating, and nausea), and lower abdominal symptoms (which included pain in the lower abdomen after a meal, when hungry, after defecation, diarrhea, and constipation).

Body mass index was calculated as the body weight (kg) divided by square of height (m) and categorized as underweight (BMI < 18.5 kg/m2), normal (BMI 18.5-22.9 kg/m2), overweight (BMI 23-24.9 kg/m2), and obese (BMI > 25 kg/m2) [13-14].

The patients then underwent upper gastrointestinal endoscopic examination and the endoscopic findings were confirmed by pathology reports. Prior to endoscopy and questionnaire administration patients were asked to give written informed consent. Approval was taken from Ethical Review Committee of the Darul Sehat hospital.

Data were stored and analyzed using IBM-SPSS version 21. Frequencies and percentages were generated for descriptive analysis whereas association of gastrointestinal symptoms and endoscopic findings was assessed using Pearson chi square test. Binary logistic regression analysis was done to module the odds ratio with 95% confidence interval (CI) for endoscopic findings (normal, not-normal); pie and bar chart was used for graphical presentation of data whereas p-value less than 0.05 was considered significant.

## Results

Out of 2148 individuals included in the study 51.8% were aged <40 years, 52.8% were male, 11.3% were underweight, 20.2% were overweight whereas 20.9% were found to be obese (Table 1).

**Table 1:** Baseline characteristics of patients (n=2148). BMI, body mass index

Variables		n	%
Age group	<= 40 years	1112	51.8
	>40 Years	1036	48.2
Gender	Male	1134	52.8
	Female	1014	47.2
BMI	Normal (<18.5)	1022	47.6
	Underweight (18.5-22.9)	243	11.3
	Over-weight (23-24.9)	433	20.2
	Obese (>25)	450	20.9

**Figure 1:**
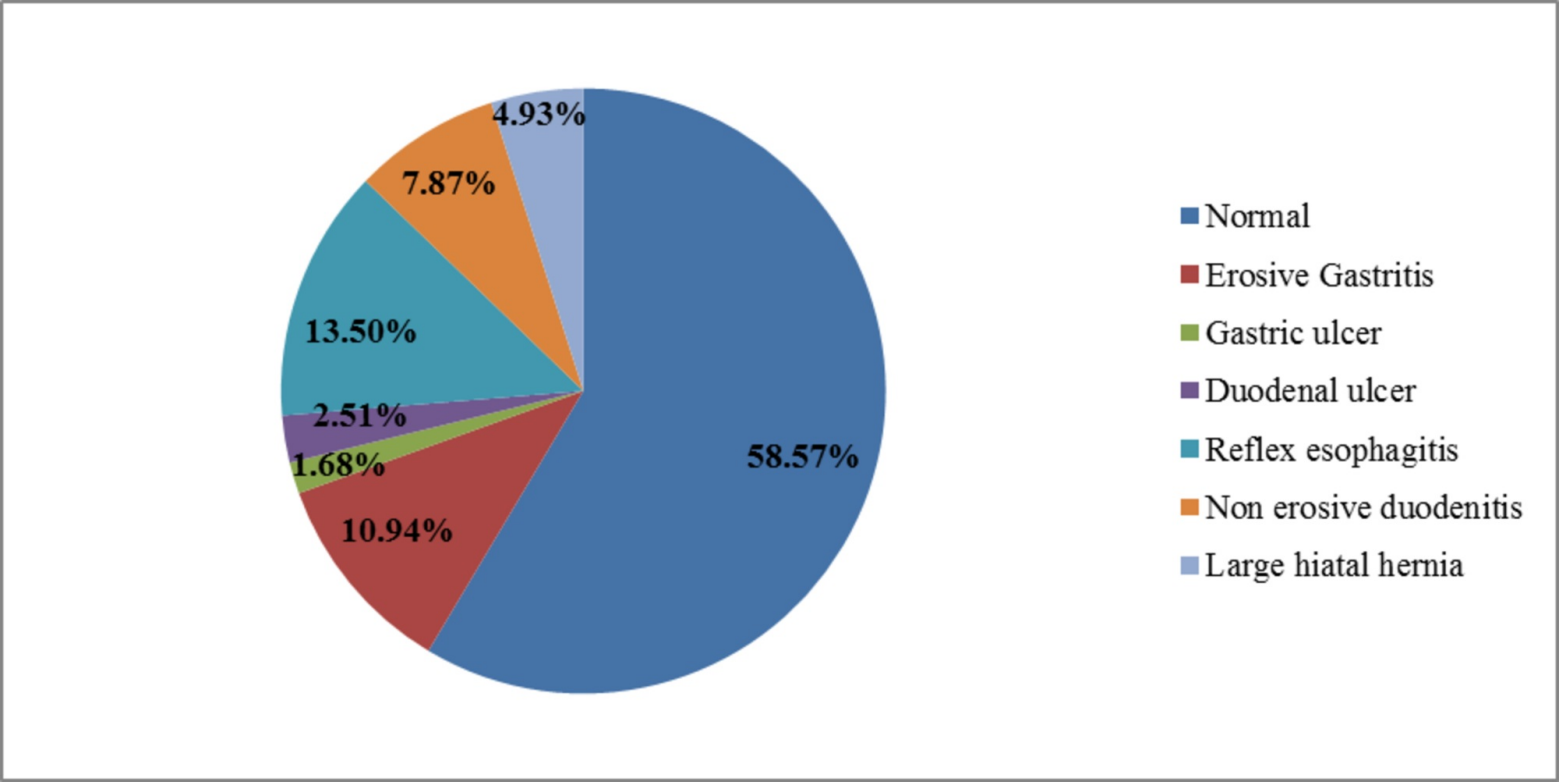
Frequency of endoscopic findings in patients (n=2148).

**Figure 2:**
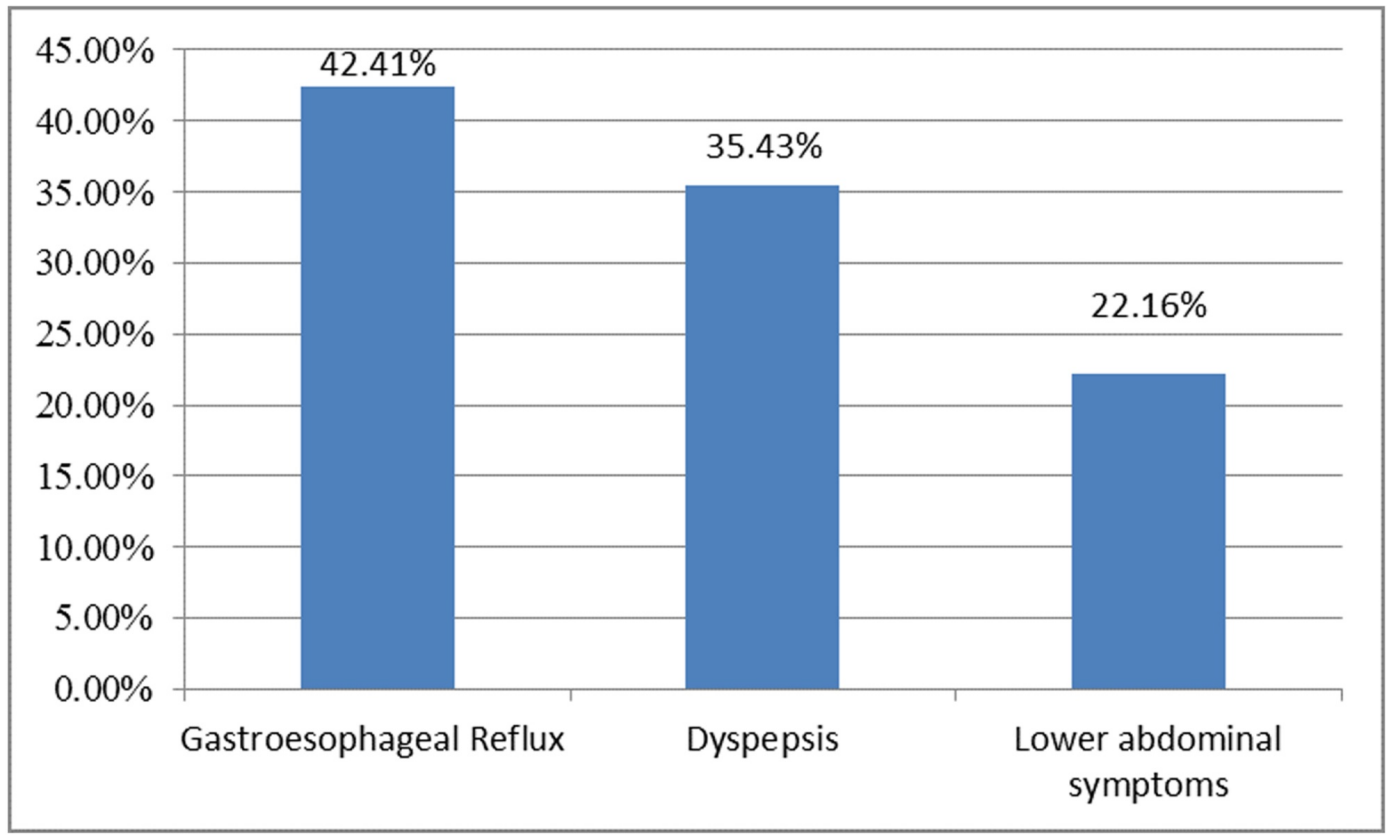
Frequency of gastrointestinal symptoms (n=2148).

The study results further revealed that both the gastrointestinal symptoms and endoscopic findings were significantly associated with the BMI (p<0.05 for both). Among gastrointestinal symptoms only the prevalence of GERD was the highest in obese individuals (48.9%) while that of dyspepsia was the highest in underweight individuals (53.1%) and that of lower abdominal symptoms was the highest in normal weight individuals (29.7%). Among endoscopic findings the prevalence of all of the erosive gastritis, duodenal ulcers, reflux esophagitis, and large hiatal hernia were found to be the highest in obese individuals (15.6%, 5.8%, 18.2%, and 8.2% respectively), that of gastric ulcers was the highest in overweight individuals (3.5%) whereas that of nonerosive duodenitis was the highest in underweight individuals (9.5%) (Figures 1-2) (Table 2).

**Table 2:** Association of gastrointestinal symptoms and endoscopic findings with BMI. BMI, body mass index; GERD, gastro esophageal reflux disease *p<0.05 was considered significant using Pearson chi square test

Variables		BMI								p-value
		Normal		Underweight		Overweight		Obese		
		n	%	n	%	n	%	n	%	
Gastrointestinal symptoms	GERD	389	38.1	113	46.5	189	43.6	220	48.9	<0.01*
	Dyspepsia	329	32.2	129	53.1	149	34.4	154	34.2	
	Lower abdominal symptoms	304	29.7	1	0.4	95	21.9	76	16.9	
Endoscopic findings	Normal	717	70.2	163	67.1	194	44.8	184	40.9	<0.01*
	Erosive gastritis	80	7.8	24	9.9	61	14.1	70	15.6	
	Gastric ulcers	12	1.2	0	0	15	3.5	9	2	
	Duodenal ulcers	4	0.4	0	0	24	5.5	26	5.8	
	Reflux esophagitis	107	10.5	33	13.6	68	15.7	82	18.2	
	Nonerosive duodenitis	66	6.5	23	9.5	38	8.8	42	9.3	
	Large hiatal hernia	36	3.5	0	0	33	7.6	37	8.2	

Moreover, binary logistic regression revealed obesity to be a significant contributor of abnormal endoscopic findings after controlling for the potential confounding effects of age and gender (adjusted odds ratio, AOR, 2.93; 95% CI, 2.35-3.65) (Table 3).

**Table 3:** OR with 95% CI for endoscopic findings. OR, odds ratio; CI, confidence interval *Odds were significant with p-value <0.05 **Model adjusted for age and gender

Risk factors	Un-adjusted OR with 95% CI	Adjusted** OR with 95% CI
Underweight	1.15 (0.85-1.55)	1.22 (0.89-1.68)
Overweight	2.9* (2.3-3.66)	0.83 (0.63-1.11)
Obese	3.4* (2.7-4.29)	2.93* (2.35-3.65)
Dyspepsia	0.96 (0.79-1.16)	0.96 (0.79-1.16)
Lower abdominal symptoms	1.01 (0.81-1.27)	0.99 (0.79-1.24)

## Discussion

The objective of the current study was to assess the relationship between increasing BMI, gastrointestinal symptoms, and upper gastrointestinal disorders in patients referred for endoscopy.

The prevalence of obesity has increased drastically all over the world. Obese patients are at danger of cardiovascular illness and unexpected losses. Obesity is reported to increase the risk of morbidity by both traditional (dyslipidemia, hypertension, and glucose dysmetabolism) and less conventional mechanisms. Less conventional risk factors include enzymes secreted by adipocytes and macrophages infiltrating adipose tissue such as adipokines, proinflammatory cytokines, and lypofibrinolytic factors that together might lead to increased oxidative stress and endothelial dysfunction thereby finally promoting atherosclerosis. At the point when established hazard variables like smoking are superimposed on the insulin resistant state of obesity they may additionally increase less conventional risk factors intensifying the existing cardiovascular issues [15].

Literature reports equivocal findings about the relationship of gastrointestinal symptoms and disorders with BMI. A systematic review reported a positive relationship between increasing BMI and the presence of GERD within the United States with an odds ratio of 1.57 for overweight and 2.15 for obese individuals [16]. In another review 79% obese people revealed reflux manifestations when contrasted with 48% normal weight people; 40% of the obese patients reported abdominal pain when contrasted with 25% normal weight patients [17]. Our outcomes are well in line with these reviews. In our study 48.9% obese individuals reported GERD symptoms, 34.2% had dyspepsia while 16.9% reported lower abdominal symptoms. Contrary to the study results, a review published in Netherlands demonstrated no significant relationship between increasing BMI and gastrointestinal symptoms [11]. This could be because of distinction in our way of life, dietary patterns, and constrained physical activity.

In Pakistan, the prevalence of gastrointestinal disorders is on the rise but only a limited number of patients are referred for endoscopy because of their poor financial status as endoscopy is a costly method and exorbitant for countless patients presenting with gastrointestinal symptoms. The relationship amongst BMI and upper gastrointestinal diseases has been explored earlier in studies published in West, however, to the best of authors’ knowledge no relevant study has previously been done in South Asia, including Pakistan. This study, therefore, is believed to be the first of its kind exploring such a relationship in Pakistan. One of the review by El-Serag et al. showed a significant increase in erosive esophagitis in people with higher BMI (26.9% in people with BMI >30 kg/m2 had reflux esophagitis when contrasted with 12.5% in people with BMI < 25 kg/m2) [18]. Another review demonstrated that esophagitis was altogether more predominant in obese (26.5%) than in normal people (9.3%) while duodenal ulcer was present in 2.5% of obese people [19]. We did, however, find reflux esophagitis as well as other gastrointestinal symptoms to be more prevalent in patients with higher BMI. In our study, 15.6% of obese patients had erosive gastritis when contrasted with 7.8% normal weight patients, 5.8% obese patients had duodenal ulcer when contrasted with 0.4% normal weight patients, and 18.2% obese patients had reflux esophagitis when contrasted with 10.5% normal weight patients. Despite asking for the medication history of the participants, unrecognized medications, for example, NSAID and medicine for the treatment of obesity (orlistat) might have confounded the observed associations.

## Conclusions

The study results revealed that some of the gastrointestinal manifestations studied occurred more frequently in obese patients. Additionally, obese patients are at danger of developing gastrointestinal disorders like reflux esophagitis, duodenal ulcers, erosive gastritis, and hiatal hernia. Keeping in view the results of this review, endeavors ought to be made to promote lifestyle modifications, exercise, and weight reduction for preventing gastrointestinal disorders.
